# Repair of pectus excavatum in a patient with an Eloesser thoracostomy window: sequential extrapleural Nuss procedure and modified Ravitch procedure

**DOI:** 10.1186/s13019-022-01914-7

**Published:** 2022-10-17

**Authors:** Jung Wook Han, Jae Jun Kim, Won Kyu Choi, Hye Yoon Jeong, Young Eun Lee

**Affiliations:** 1 Department of Thoracic and Cardiovascular Surgery, Uijeongbu St. Mary’s Hospital, College of Medicine, The Catholic University of Korea, Geumo-dong, Gyeonggi-do 480-717 Uijeongbu, Republic of Korea; 2 Department of Anesthesia and Pain Medicine, Uijeongbu St. Mary’s Hospital, College of Medicine, The Catholic University of Korea, Seoul, Republic of Korea

**Keywords:** Pectus excavatum, Eloesser window operation, Nuss procedure, Ravitch procedure

## Abstract

A 28-year-old man with a history of tuberculous empyema and pectus excavatum visited our hospital for progressive dyspnea and leg edema. The patient had undergone an Eloesser window operation for repetitive pleuro-cutaneous fistula due to chronic tuberculous empyema in the left thorax one year prior. Chest computed tomography demonstrated severe compression of the right ventricle and inferior vena cava and chronic empyema with the Eloesser window in the left thorax. Because conservative treatment had failed, the patient underwent a total extrapleural Nuss procedure, resulting in marked relief of compression and complete resolution of leg edema and congestive hepatopathy. However, he required ventilation support due to carbon dioxide retention. Therefore, the patient underwent a modified Ravitch procedure and was weaned off ventilation support. Herein, we represent the first report of a sequential extrapleural Nuss procedure and a modified Ravitch procedure in a patient with chronic tuberculous empyema with an Eloesser window.

## Background

Pectus excavatum is the most common chest wall deformity, and the Nuss procedure has become the preferred option for its correction due to its minimal invasiveness [1, 2]. However, the standard Nuss procedure is not applicable in all circumstances, and modification of the standard Nuss procedure or selection of the Ravitch procedure is needed in some cases [3, 4]. Herein, we represent the first report of a sequential extrapleural Nuss procedure and a modified Ravitch procedure in a patient with chronic tuberculous empyema with an Eloesser window.

## Case presentation

**Fig. 1:**
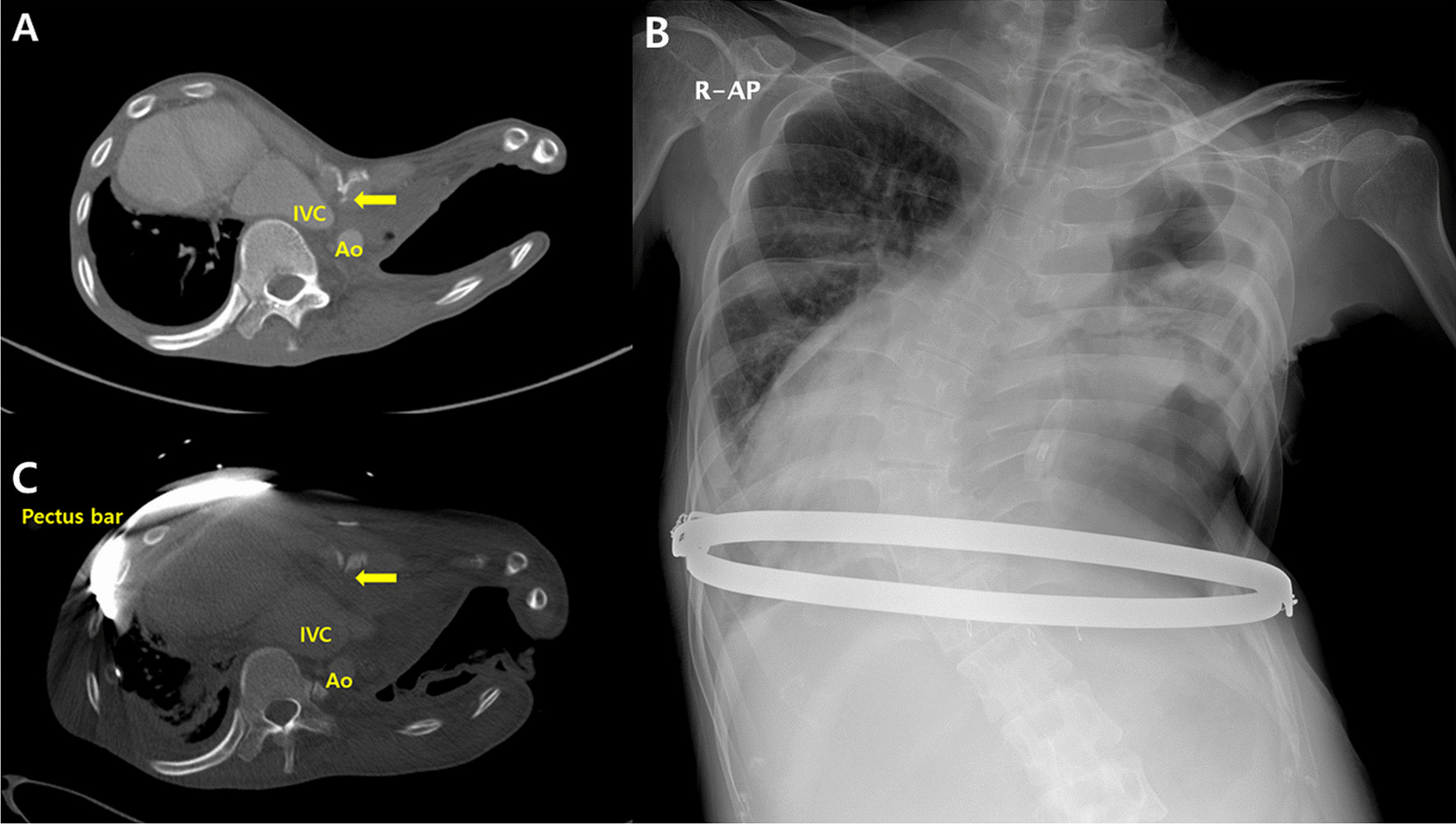
Images before and after the extrapleural Nuss procedure. **A** Initial chest computed tomography shows severe chest wall depression, compression of the inferior vena cava, spinal scoliosis, dextrocardia, and chronic left empyema with the Eloesser window. **B** A simple chest x-ray at discharge shows the extrapleural Nuss procedure with the sandwich technique. The patient had a tracheostomy for ventilator support. **C** A one-month postoperative chest computerized-tomography reveals a marked relief of compression on the heart and inferior vena cava by the depressed chest wall (IVC: inferior vena cava, Ao: descending aorta, arrow: most depressed point)

A 28-year-old man was referred for progressive dyspnea and edema in both legs. His history included pectus excavatum and an Eloesser window operation for repetitive pleuro-cutaneous fistula due to chronic tuberculous empyema in the left thorax one year prior. Physical examination revealed a severe chest wall depression and generalized pitting edema that was particularly severe in his bilateral lower extremities. Chest computed tomography (CT) showed a severe chest wall depression, compression of the inferior vena cava and right ventricle, spinal scoliosis, dextrocardia, and chronic left empyema with an Eloesser window (Fig. 1A). Deep vein thrombosis was not observed in either lower extremity, and abdominal CT and ultrasonography revealed hepatic congestion. Laboratory findings revealed hepatic abnormality (total bilirubin 6.6 mg/ dl, direct bilirubin 3.1 mg/dl, AST 371 U/L, ALT 225 U/L, and ALP 413 U/L). An echocardiogram showed hypokinesis of the left ventricle anterior wall and decreased volume of the right ventricle, but left ventricle systolic function was not impaired (ejection fraction 50%). The Eloesser window operation was determined to aggravate the pectus excavatum, which caused his conditions.

**Fig. 2:**
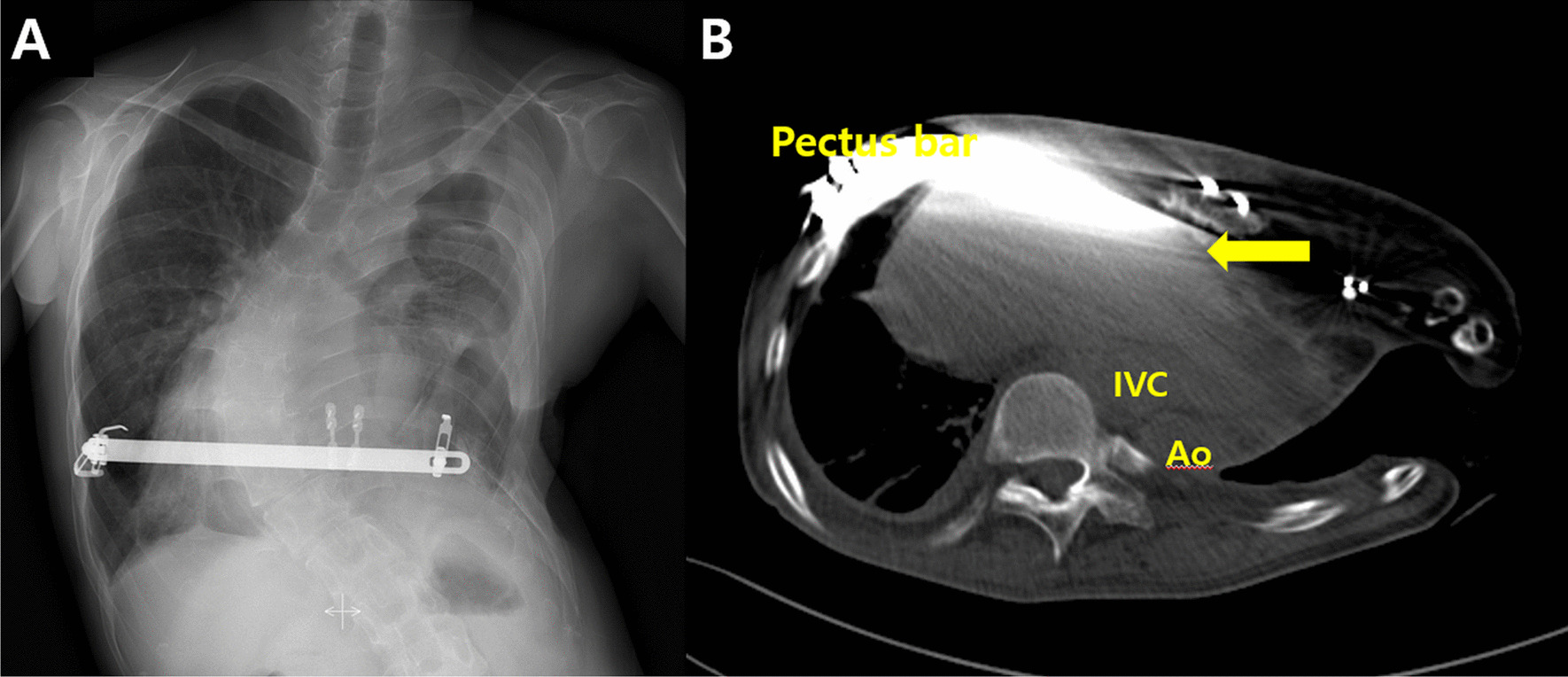
Images after the modified Ravitch procedure. **A** A simple chest x-ray shows the modified Ravitch procedure with a pectus bar. The tracheostomy had been removed. **B** Chest computerized-tomography six months after the Ravitch procedure shows successful relief of compression on the heart and inferior vena cava by the depressed chest wall (IVC: inferior vena cava, Ao: descending aorta, arrow: most depressed point)

The patient initially was treated conservatively. However, hepatic dysfunction and symptoms (dyspnea and leg edema) did not resolve. Therefore, we decided to proceed with surgical intervention to manage pectus excavatum and chose the Nuss procedure for its minimal invasiveness. Due to the empyema with the Eloesser window, a modification of the standard Nuss procedure was needed. After sternal elevation for safety, small incisions were created at the subxiphoid area and at the bilateral mid-axillary lines, avoiding the left Eloesser window. A plane between the sternum and the parietal pleura was created, and a bilateral extrapleural space between the two bilateral hinge points was created using sharp and blunt dissection through the subxiphoid and bilateral mid-axillary line skin incisions. A chest tube (20 French) was passed through the space between the two bilateral hinge points. A bent pectus bar was inserted by following the chest tube as a guide while simultaneously removing the chest tube. Turning the convex pectus bar elevated the depressed chest wall. Another bent bar was inserted through the presternal route to fixate the inserted bar, and both ends of the two pectus bars (internal and external) were tied with wires bilaterally (the sandwich technique) [5]. All procedures were conducted under direct thoracoscopic vision. Two weeks after surgery, hepatic dysfunction and lower leg edema were completely resolved. However, a tracheostomy was performed due to prolonged ventilation support. Although the patient did not require oxygen supply (approximately 70 mm Hg at room air), ventilation weaning failed due to carbon dioxide retention (up to 120.1 mm Hg). The patient tolerated being without a ventilator for hours, but he could not completely be weaned from the ventilator. He was discharged on the 21st postoperative day with a tracheostomy and ventilator support (Fig. 1B). A one-month postoperative chest CT revealed marked relief of compression of the heart and inferior vena cava by the depressed chest wall (Fig. 1C). Despite respiratory rehabilitation for six months, the patient could not be weaned completely from the ventilator. Chest wall movement was found to be restricted during respiration, and we believed that ventilator support was needed due to restriction of the chest wall movement caused by the metal bars used in the Nuss procedure with the sandwich technique. Therefore, we altered the repair method to the modified Ravitch procedure using a short pectus metal bar. The patient was able to be weaned from ventilator support completely after one month of respiratory rehabilitation (Fig. 2). The patient has been followed up without any complications.

## Discussion

Other than cardiopulmonary impairment, pectus excavatum has been reported as a rare cause of syncope, leg edema, and dysphasia [6]. The Nuss procedure has become a preferred option for correction of pectus excavatum worldwide, due to its excellent functional and cosmetic outcomes [1, 2]. However, the standard Nuss procedure is not applicable in all circumstances, and modification or the Ravitch procedure is necessary in some cases [3, 4].

In the present case, the Eloesser window operation was considered to aggravate pectus excavatum, which caused congestive hepatopathy and bilateral leg edema through compression of the right ventricle and inferior vena cava [7]. Since conservative treatments had been unsuccessful, surgical intervention was planned. The aim of surgical repair was to improve cardiac function and congestive hepatopathy by relieving the compression on the heart and inferior vena cava. The patient’s empyema with the Eloesser window forbade the standard Nuss procedure (through the intrapleural route). Therefore, a modification (through the extrapleural route) was performed, and several previous studies have reported that the extrapleural Nuss procedure is a safe and less traumatic procedure compared to the standard Nuss procedure [3, 4]. Although, there were a few injuries to the right pleura during the procedure, there was no injury or perforation in the left pleura due to its altered nature (thickened due to empyema). In addition, the use of a small subxiphoid incision made the procedure safer and more feasible. We chose the sandwich technique because empyema with the Eloesser window restricted the usage of usual instruments for fixation to the ribs [5].

Compression on the heart and inferior vena cava was markedly relieved, and congestive hepatopathy was completely resolved. However, the patient required ventilator support due to carbon dioxide retention even though he tolerated no ventilator support for hours. The sandwich technique was considered to impair his respiratory function because the pectus metal bars restricted chest wall movement (8). Since the patient was in very poor condition including severe spinal scoliosis, dextrocardia, and chronic left empyema with the Eloesser window, even mild restriction of chest wall movement by pectus metal bars was assumed to cause respiratory distress. Since ventilator weaning ultimately failed, the modified Ravitch procedure was performed with a short pectus bar, avoiding entry into the empyema space. The patient was weaned off ventilator support completely one month after the Ravitch procedure. The present case report suggests followings. The first, we can repair pectus excavatum through the modification of the Nuss procedure in a patient with an Eloesser thoracostomy window. The second, the sandwich method can restrict chest wall movement in some conditions because the Nuss procedure is usually considered to not cause greater restriction of chest wall movement compared to the Ravitch procedure [1, 8].

## Conclusion

We present a case of successful repair of pectus excavatum with the Eloesser window through a sequential extrapleural Nuss procedure and a modified Ravitch procedure. The Nuss procedure should be adapted to certain circumstances, and the pectus metal bar can cause respiration impairment in some conditions.

## Data Availability

Not applicable.
